# Polycystic Ovary Syndrome: A Comprehensive Review of Pathogenesis, Management, and Drug Repurposing

**DOI:** 10.3390/ijms23020583

**Published:** 2022-01-06

**Authors:** Hosna Mohammad Sadeghi, Ida Adeli, Daniela Calina, Anca Oana Docea, Taraneh Mousavi, Marzieh Daniali, Shekoufeh Nikfar, Aristidis Tsatsakis, Mohammad Abdollahi

**Affiliations:** 1 Toxicology and Diseases Group (TDG), Pharmaceutical Sciences Research Center (PSRC), The Institute of Pharmaceutical Sciences (TIPS), Tehran University of Medical Sciences, Tehran 11369, Iran; hosnams1379@gmail.com (H.M.S.); adeli_ayda@yahoo.com (I.A.); st-Mousavi@student.tums.ac.ir (T.M.); marziehdaniali75@gmail.com (M.D.); 2 Department of Toxicology and Pharmacology, Faculty of Pharmacy, Tehran University of Medical Sciences, Tehran 11369, Iran; 3 Department of Clinical Pharmacy, University of Medicine and Pharmacy of Craiova, 200349 Craiova, Romania; 4 Department of Toxicology, Faculty of Pharmacy, University of Medicine and Pharmacy, Petru Rares, 200349 Craiova, Romania; daoana00@gmail.com; 5 Department of Pharmacoeconomics and Pharmaceutical Administration, Faculty of Pharmacy, Tehran University of Medical Sciences, Tehran 11369, Iran; shekoufeh.nikfar@gmail.com; 6 Personalized Medicine Research Center, Endocrinology and Metabolism Research Institute, Tehran University of Medical Sciences, Tehran 11369, Iran; 7 Evidence-Based Evaluation of Cost-Effectiveness and Clinical Outcomes Group, Pharmaceutical Sciences Research Center (PSRC), The Institute of Pharmaceutical Sciences (TIPS), Tehran University of Medical Sciences, Tehran 11369, Iran; 8 Department of Analytical and Forensic Medical Toxicology, Sechenov University, 119991 Moscow, Russia; aristsatsakis@gmail.com; 9 Department of Forensic Sciences and Toxicology, Faculty of Medicine, University of Crete, 71003 Heraklion, Greece; 10 Laboratory of Toxicology, Medical School, University of Crete, 70013 Heraklion, Greece

**Keywords:** polycystic ovary syndrome, hyperandrogenism, insulin resistance, molecular mechanisms, management, repurposing drugs

## Abstract

Polycystic ovary syndrome (PCOS) is an endocrine-gynecology disorder affecting many women of childbearing age. Although a part of the involved mechanism in PCOS occurrence is discovered, the exact etiology and pathophysiology are not comprehensively understood yet. We searched PubMed for PCOS pathogenesis and management in this article and ClinicalTrials.gov for information on repurposed medications. All responsible factors behind PCOS were thoroughly evaluated. Furthermore, the complete information on PCOS commonly prescribed and repurposed medications is summarized through tables. Epigenetics, environmental toxicants, stress, diet as external factors, insulin resistance, hyperandrogenism, inflammation, oxidative stress, and obesity as internal factors were investigated. Lifestyle modifications and complementary and alternative medicines are preferred first-line therapy in many cases. Medications, including 3-hydroxy-3-methyl-3-glutaryl-coenzyme A (HMG-CoA) reductase inhibitors, thiazolidinediones, sodium-glucose cotransporter-2 inhibitors, dipeptidyl peptidase-4 inhibitors, glucose-like peptide-1 receptor agonists, mucolytic agents, and some supplements have supporting data for being repurposed in PCOS. Since there are few completed clinical trials with a low population and mostly without results on PCOS repurposed medications, it would be helpful to do further research and run well-designed clinical trials on this subject. Moreover, understanding more about PCOS would be beneficial to find new medications implying the effect via the novel discovered routes.

## 1. Introduction

Polycystic ovary syndrome (PCOS) is a heterogeneous endocrine disorder that impacts many women of the reproductive age worldwide [1]. This syndrome is often associated with enlarged and dysfunctional ovaries, excess androgen levels, resistance to insulin, etc. [2]. It is estimated that approximately every 1 in 10 women face PCOS before menopause and struggle with its complications [3]. 

Although the high ratio of luteinizing hormone (LH) to follicle-stimulating hormone (FSH) and increased frequency of gonadotropin-releasing hormone (GnRH) is known as the underlying causes of PCOS [4], the exact etiology and pathology have not been comprehensively well-known [4,5]. Evidence suggests the role of different external and internal factors, including insulin resistance (IR), hyperandrogenism (HA), environmental factors, genetic, and epigenetics. In addition, it is worth mentioning that PCOS increases the risk of further complications like cardiovascular diseases [5,6], type 2 diabetes mellitus [5,6], metabolic syndrome [6], depression, and anxiety [7].

To manage this condition, the most crucial step is to lose at least 5% of the weight; therefore, having a regular exercise plan and fat and sugar-free diets are also recommended to every woman with PCOS. Furthermore, in some cases, taking complementary and alternative medicine strategies with or without other treatments is preferable due to their prior beliefs, lower costs, etc. 

Physicians tend to use (combined) oral contraceptives, antiandrogen agents, insulin sensitizers, and ovulation inducers [4]. Up until today, there is no United States Food and Drug Administration (USFDA) approved medication specifically for PCOS, and all mentioned medications are used off-label [8]. Apart from the essential need for improvement in the research and development of new drug molecules and new drug discovery, novel medications could be found with drug repurposing methods [9]. On this very spot, there are plenty of medications, previously approved by USFDA for indications rather than PCOS; and, today, there is a desire to implement them as the therapeutic options in the management of PCOS.

These agents vary from anti-diabetic medications such as pioglitazone, empagliflozin, sitagliptin, liraglutide to 3-hydroxy-3-methyl-3-glutaryl-coenzyme A (HMG-CoA) reductase inhibitors like simvastatin and atorvastatin, as well as mucolytic drugs like N-acetyl cysteine. 

Given that PCOS is a growing issue that is unfortunately followed by many unwanted complications and that available methods and medications are not 100% effective, it is essential to investigate its pathogenesis and find out new pharmacological targets carefully. This could be done through repositioning approaches, saving time and cost. 

This review discusses PCOS’s definition, diagnosis, and etiology, focusing on the pathogenesis and management of this syndrome. Internal and external factors contributing to PCOS have been comprehensively studied, and several commonly prescribed medications with their complete drug information are provided. Subsequently, a couple of repurposed medications are mentioned thoroughly, reviewing the related clinical trials over the past five years. 

## 2. Methods

PubMed, Google Scholar, ScienceDirect, TRIP database, and UpToDate were comprehensively searched for publications including PCOS relevant keywords in different areas, focusing on the new ones (since 2016) and excluding those with a language rather than English or animal studies. In addition, Clinicaltrials.gov was searched to find data about completed or running trials of repurposed drugs in PCOS over the past five years. 

## 3. Diagnosis

PCOS is among the conditions that cannot be diagnosed with basic diagnostic tests, including blood tests, culture, and biopsy; thus, there is no certain test for PCOS diagnosis. Differential diagnosis is called excluding the relevant disorders according to the symptoms and narrowing the choices. In order to establish a differential diagnosis for PCOS, hyperprolactinemia, thyroid disease, Cushing’s syndrome, and hyperplasia of adrenal should be excluded based on the associated investigations [10,11]. Although considering past medical history, weight changes, and symptoms of insulin resistance might be helpful, pelvic examination, a transvaginal ultrasound, and measuring the level of hormones are among the most frequently recommended investigations [12]. According to the National Health Service (NHS), irregular or infrequent periods, high levels of androgenic hormones or symptoms, and scans showing polycystic ovaries are the specified criteria for PCOS [13]. In addition, Rotterdam PCOS diagnostic criteria in adults are the most commonly used method. In an ultrasound, the presence of two clinical or biochemical hyperandrogenism, ovulatory dysfunction, or polycystic ovaries would finalize a PCOS diagnosis [14].

## 4. Etiology and Risk Factors

### 4.1. External Factors

#### 4.1.1. Epigenetic Mechanism

Epigenetic refers to inheritable alterations in genome and gene expression without any changes in DNA sequence [15,16]. These changes involve adding or omitting chemical components on DNA or histone [17]. Increased LH activity is a seen phenomenon in PCOS women. It may relate to the problems in follicle development and HA, which are common among PCOS patients [18]. LH/choriogonadotropin receptor (LHCGR) is responsible for the steroidogenesis process in theca cells [19]. This receptor hypomethylation leads to higher gene expression and sensitivity to LH [18,20]. 

A study on PCOS patients approved that hypomethylated sites are related to overexpression of LHCGR [15,19] on theca cells surface [19]. In addition, epoxide hydrolase 1 (EPHX1) is an active enzyme in degrading aromatic compounds [15,19,21]. Its gene promoter hypomethylation [15,19] increases enzyme expression [15]. Overproduction of EPHX1 reduces the transformation of testosterone to estradiol, which can contribute to PCOS [15]. Furthermore, peroxisome proliferator-activated receptor gamma (PPAR-γ) plays a role in ovaries’ function [15,18,19,22]. Hypermethylation of PPARγ, hypomethylation of nuclear co-repressor 1 [19,22], and alteration in acetylation of histone deacetylase 3, for which both are PPARγ co-repressors [15], are observed in PCOS patients showing HA [15,19,22]. These alterations were noticed in PCOS women’s granulosa cells [18,23].

#### 4.1.2. Environmental Toxicants

The United States Environmental Protection Agency (USEPA) defines endocrine-disrupting chemical (EDC) as “an exogenous agent that interferes with the synthesis, secretion, transport, binding, action, or elimination of natural hormones in the body that are responsible for the maintenance of homeostasis, reproduction, development and/or behavior” [24].

EDCs may act as hormones’ agonists or antagonists in binding to their receptors [25]. EDCs are almost parts of everything we use in our daily life [21]. Their structures consist of phenols or halogens like chlorine and bromine, so they imitate steroid hormones’ actions [21]. Studies have approved the higher serum concentration of EDCs in PCOS suffering women [21,26]. Prolonged and continuous exposure to EDCs from prenatal to adolescence can cause susceptibility to PCOS [21,27]. 

As an example, bisphenol A (BPA) BPA is a synthetic compound used in polycarbonate plastics, epoxy resins [25,28], dental filling, food and drink packages [25], baby bottles, and polyvinyl chloride (PVC) [28], which affects metabolism through different pathways. BPA directly affects oogenesis [29] by interacting with estrogen receptor (ER) α and β, non-classical membrane ER, and G-protein coupled receptor 30 (GPCR30) [21,28,29]. It also triggers androgen secretion and restrains testosterone catabolism in theca cells [21,29]. 

Another effect of BPA on interstitial theca cells is the overproduction of androgens by dysregulation of 17β-hydroxylase (P450c17) [28,30], cholesterol side-chain cleavage enzyme (P450scc), and steroidogenic acute regulatory protein [30]. BPA’s influence on granulosa cells refers to reducing the expression of aromatase enzyme and production of estrogen [21,29]. Lastly, it disturbs the intrafollicular environment and damages the oocyte development and maturation [21,29]. BPA’s indirect effect on HA involves downregulation of testosterone 2a-hydroxylase and testosterone 6b-hydroxylase enzymes in liver level, and thus a higher concentration of testosterone [30,31]. 

In addition, BPA is a potent ligand for sex hormone-binding globulin (SHBG) and replaces testosterone; thereby, free testosterone concentration increases. Androgen and BPA have a two-way relation; high androgen inactivates the uridine diphosphate-glucuronosyl transferase enzyme and reduces BPA clearance in the liver. This process causes a high concentration of free BPA in blood and worsens its negative effects on the ovaries [21,29,30,31]. 

Additionally, it is believed that BPA may act as an obesogen [28,30]. Its obesogenic influence includes upregulation of adipogenesis-related genes [30], stimulation of adipocytes differentiation [28,30], potentiation of the accumulation of lipid in cells incorporated in medical syndrome, and triggering the conversion of target cells to adipocytes via phosphatidylinositol 3-kinase pathway [30]. 

Adipogenesis due to BPA happens because of the activation of the glucocorticoid receptor. Activation of the receptor upregulates the enzyme involved in the conversion of cortisone to cortisol, thus inducing adipogenesis [28]. Moreover, BPA prompts the release of interleukin-6 (IL-6) and tumor necrosis factor α (TNF-α) [30,31], both involving adiposity and IR [30]. In addition, it restrains the release of adiponectin [28,29,30,31] and the beneficial compound in protecting against IR [28,30,31].

It can also change glucose homeostasis [28,29,31] by directly influencing the pancreatic cells [29]. BPA causes a chronic increase in insulin and further IR in long exposure [30] by affecting the mitochondrial activity and metabolic pathways of β-pancreatic cells [28]. BPA reduces glucagon secretion by inhibiting the intracellular calcium ion fluctuating pattern with a lack of glucose condition [30].

Advanced glycation end products (AGEs), also called glycotoxins, are another chemical group affecting body health. AGEs are pro-inflammatory molecules [21,23,29,32] that interact with their surface receptor called RAGE (receptor for AGE) [21,23,29] and stimulate pro-inflammatory pathways and oxidative stress [21,23,29,32]. AGEs can be absorbed into the body as exogenous compounds or derived from nonenzymatic glycation and oxidation of proteins and lipids [21]. Increased concentration of AGEs in serum has been detected in PCOS patients [21]. AGEs interrupt pre-ovulatory follicles growth via ERK1/MAPK pathway and damage follicles by oxidative stress caused by interaction with RAGEs [21]. This interaction increases intracellular inflammatory molecules [21]. 

In vitro studies on 3T3-L1 cell lines showed that glycotoxins are likely to trigger adipogenesis [21]. On the other hand, a higher body mass index corresponds to a lower extent of soluble RAGEs, which is responsible for glycotoxin clearance and deposition of AGEs in the reproductive system, especially in ovaries [21,29]. This bilateral relation worsens inflammatory processes and metabolic syndrome in PCOS [21]. AGEs also play a role in IR [21,29]. These compounds disrupted glucose transport in the human granulosa KGN cell line [21] and reduced glucose uptake by adipocytes in previous research [21,29]. They also involve IR by causing oxidative stress, inflammation, and glycation of proteins, which considerably diminishes insulin sensitivity [21]. Moreover, increased concentration of AGEs changes the insulin signaling pathway and interferes with glucose transporter 4 (GLUT-4) translocation [23].

#### 4.1.3. Physical and Emotional Stress

Although there is minimal information on the role of stress in PCOS, it is known that PCOS possesses adverse effects on self-esteem and mental health. Chronic stress results in hypertrophy and hyperplasia of adipocytes. This phenomenon happens as a result of glucocorticoids’ effect on pre-adipocytes maturation. Chronic stress is also associated with adipokine secretion, attraction, and activation of stromal fat immune cells [33]. 

In addition, it is responsible for making an inflammatory condition by leading to high levels of inflammatory cytokines like IL-6 and TNF-α, along with disrupting oxidant-antioxidant balance [33]. In addition, chronic stress plays a vital role in IR. 

Stress triggers the hypothalamic-pituitary-adrenal (HPA) axis to release cortisol [34,35]. Cortisol leads to IR by stimulating visceral fat accumulation, gluconeogenesis, and lipolysis [35]. Moreover, cortisol arouses glucose production in the liver [35]. Stress is also involved in enhancing insulin levels [34]. Other stress influences on PCOS may refer to inference with anti-mullerian hormone (AMH) and changing sex hormone levels [34,35]. 

#### 4.1.4. Diet

Although nutrition contributions to PCOS is unclear, studies showed a relationship between some nutrient levels and PCOS indices. 

Saturated fatty acids (SFAs) intake plays a role in PCOS by producing an inflammatory status [36] and reducing insulin sensitivity [37]. Taking SFAs induces inflammation by triggering an increase in TNF-α level in circulation and expressing a specific cytokine suppressor [36]. 

Vitamin D deficiency may exacerbate PCOS [37,38] or the comorbidities induced by PCOS [38]. Calcitriol upregulates insulin receptors at mRNA and protein levels. It also increases insulin sensitivity directly and indirectly. The direct effect occurs by activating PPAR-δ, the involved receptor in fatty acids metabolism in adipose tissue and skeletal muscle. The indirect impact is the regulation of intracellular calcium, which is vital for insulin-mediated signaling in fat and muscle [38]. On the other hand, vitamin D deficiency may result in insulin resistance by causing an inflammatory response [37,39]. Furthermore, vitamin D downregulates the AMH promoter [39].

### 4.2. Internal Factors

#### 4.2.1. Insulin Resistance

IR means an insufficient cells response to insulin [40]. IR is independent of patients’ adiposity, body fat topography, and androgen levels [18,41]; i.e., it has been reported in lean patients as well [18,42]. It should be mentioned that IR is tissue-selective in PCOS women [18,43], although skeletal muscles [18,43,44], adipose tissue, and liver lose their sensitivity to insulin, adrenal glands [18,43], and ovaries remain sensitive [18,28,43,45].

Insulin directly triggers androgens production in ovarian theca cells [32,44,46,47,48] and grow [48]. Insulin effectively stimulates ovarian follicle growth and hormone secretion by stimulating its receptors in the follicle membrane cells [49]. It also triggers ovarian P450c17 [18,23,50] and P450scc enzyme activity to promote ovarian steroidogenesis [18,51] and increases them with the synergistic effect of chorionic gonadotropin [52]. This hormone, as well as insulin-like growth factor 1 (IGF-1) [18], synergizes with luteinizing hormone [18,45]. Hyperinsulinemia increases LH-binding sites and androgen-producing response to LH [44]. LH and insulin interaction enhance steroidogenic acute regulatory enzyme and CYP450c17 mRNA expression [52,53]. CYP450c17 is involved in androgen production [23,44]. Likewise, IR independently enhances CYP17A1 activity, the productive enzyme in androstenedione and testosterone production [52]. 

On the other hand, hyperinsulinemia reduces hepatic SHBG [18,32,40,49,52,54,55,56], increasing free testosterone levels in blood [18,32,52,54,56]. In addition, hyperinsulinemia inhibits IGF-1 binding protein production in the liver. IGF-1 is responsible for triggering the production of androgens in thecal cells. Inhibition of the production of IGF-1 binding proteins leads to a higher concentration of this substance in blood circulation and then higher production of androgens in thecal cells [18,46]. Moreover, IGF-1 upregulation decreases a specific miRNA and thus accelerates granulosa cells apoptosis and inhibits folliculogenesis [52]. HA [46] and hyperinsulinemia [45,46,57] both play a role in stopping follicles growth [45,46]. This stoppage is attributed to menstrual irregularity, anovulatory sub-fertility, and amassing of immature follicles [46].

Furthermore, hyperinsulinemia contributes to PCOS by affecting the pituitary gland. Excessive insulin stimulates its receptors in the pituitary gland to release LH [49]. Accumulation of insulin stimulates GnRH and LH pulse secretion via influencing both amplitude and frequency [23]. Insulin’s indirect effect on PCOS is augmented by pituitary gonadotropin sensitivity to GnRH [18], and hyperinsulinemia increases GnRH neuron activity [58].

The insulin’s influence on adipose tissue and inflammation is another essential PCOS pathogenesis topic. Insulin stimulates adipogenesis and lipogenesis and inhibits lipolysis [42], resulting in fat accumulation [44]. IR leads to enhanced plasma levels of free fatty acids (FFAs), affecting the liver and adipose tissue [32]. Moreover, IR causes a reduction in omentin level independent of the patient’s body mass index (BMI). In addition, hyperglycemia can lead to inflammation by producing TNF-α from mononuclear cells (MNCs) [50]. 

#### 4.2.2. Hyperandrogenism

Generally, hyperandrogenism (HA) reduces the SHBG level, leading to a higher concentration of free testosterone [18,59]. It was observed that PCOS women have higher concentrations of testosterone in plasma which can convert to estrone in adipose tissue. Increased alteration of estrone to estradiol affect follicle growth and increases the LH to FSH ratio causing ovulatory dysfunction [23]. 

HA can result in AMH upregulation, which inhibits ovulation and the development of follicles by a different mechanism. Furthermore, the IGF-II level is negatively related to androgen levels, and HA reduces IGF-II in follicular fluid. IGF-II positively relates to follicle diameters and estradiol concentration in follicular fluid [23]. In addition, HA increases LH indirectly [58,60]. Estradiol and progesterone are responsible for GnRH and LH secretion via negative feedback [58,61,62]. HA disrupts the negative feedback on secretion [18,23,61,62] resulting in increased LH levels [18,62]. Interaction of androgen and its receptor interferes with progesterone receptor transcription. Moreover, this receptor is involved in converting high levels of androgens to compounds that modulate the gamma-aminobutyric acid A (GABA_A_). Modulation of the GABA_A_ receptor triggers GnRH neurons and weakens the response to negative progesterone feedback [58]. In addition, it is assumed that androgens might decrease hepatic nuclear factor-4α (HNF-4α) levels by inhibiting lipid synthesis. HNF-4α stimulates SHBG expression by binding to its promoter [63].

HA contributes to other influential factors of PCOS, including IR, inflammation, and oxidative stress. 

HA aggravates IR via different routes; it reduces the insulin sensitivity, expression of GLUT-4 and inhibits insulin degradation in the liver [23,32]. Moreover, HA increases a type of skeletal muscle fibers that have low insulin sensitivity [32]. On the other hand, HA worsens central adiposity, which is involved in IR [23,32]. Additionally, it was observed that testosterone increases inflammatory chemicals such as lipopolysaccharide-induced IL-6 in 3T3-L1 adipocytes by activating some signaling pathways [64]. One way androgen results in oxidative stress is by increasing MNC sensitivity to glucose and aggravating glucose-stimulated oxidative stress [65]. It is worth mentioning that dehydroepiandrosterone as an androgen decreases interferon-γ (IFN-γ), an essential regulator in normal ovarian physiology and cell function [64]. 

In addition, it should be mentioned that studies on PCOS women approved the resemblance of their fatty tissue to men, and hence the effect of HA on adipose tissue dysfunction [8]. In addition, HA is a cause of adipocyte hypertrophy and consequential damages to adipokine secretion [55].

#### 4.2.3. Inflammation

Appropriate inflammation is a vital cause of oocyte growth and ovulation [66]. However, high levels of white blood cell [46,66], C-reactive protein (CRP) [4,46,50,66,67], and other inflammatory biomarkers in peripheral blood are associated with PCOS [4,46,66,67,68]. Inflammation is a cause of HA [44,69]. TNF-α is a pro-inflammatory chemical that can worsen IR. Contribution to IR happens due to interference of pro-inflammatory molecules with insulin signaling pathways [32,67] and reduction of GLUT-4 expression [23]. Some studies showed that the insulin receptor substrate (IRS) serine residue phosphorylation inhibits insulin receptor signaling [32,70]. This phenomenon results in the prevention of GLUT-4 translocation and glucose reuptake [70]. In addition, TNF-α showed the ability to prompt theca cells proliferation in vitro [71]. Furthermore, IL-1 hinders the FSH and LH receptors. Inhibition of these receptors leads to inhibition of follicular development and ovulation [66]. Both TNF-α and IL-1β inhibit activation of HNF-4α by different mechanisms [23]. In addition, NLRP3 inflammasomes induce follicular pyroptosis, ovarian fibrosis, and disturbance of follicular formation [66]. An increase in CRP level is another cause of IR in insulin-sensitive tissues. IR occurs because of increased pro-inflammatory factors secreted by the liver and monocytes. CRP stimulates this increase in secretion [72]. Moreover, another study approved the higher-than-normal level of IL-6 mRNA in granulosa cells [66].

#### 4.2.4. Oxidative Stress

Oxidative stress (OS) is an imbalance between pro-oxidants and antioxidants [71,72,73]. Oxidative molecules include different chemicals such as reactive oxygen species (ROS) [73,74,75] (e.g., O^2−^, H_2_O_2_, and OH^−^) [76] and reactive nitrogen species (RNS) [74,75]. ROS plays a role in different mechanisms like signaling pathways [71,73,76], cell growth [71,73], and differentiation, as well as RNS [73]. RONS also acts on ovaries functions like steroidogenesis [67,77] and affects neurons responsible for feeding behavior to induce hunger [71]. Overproductions of oxidative chemicals cause various damage to vital molecules such as lipids, proteins, and DNA [73,74,75,77]. 

Increased OS has been seen in PCOS patients in different studies [74,78,79]. Increased levels of OS activate the nuclear factor-kappa B (NF-κB) [72,75]. NF-κB is involved in inflammatory pathways [75] and affects the production of pro-inflammatory cytokines like TNF-α and IL-6 [72,80]; the effect in IR and PCOS was explained above. A high level of OS also increases the release of TNF-α [77]. On the other hand, increased OS actuates some protein kinases that trigger serine/threonine phosphorylation instead of normal tyrosine phosphorylation of IRS. Thus, the insulin signaling pathway is inhibited, and OS leads to IR [67]. OS also plays a role in obesity. It increases mature adipocyte size and consequently stimulates pre-adipocyte proliferation and adipocyte differentiation. OS also imposes a major effect on obesity [71].

#### 4.2.5. Obesity

Obesity is a key in low-grade chronic inflammation [72]. Accumulation of adipocytes in visceral fat leads to hypoxia and consequent necrosis, which causes inflammatory cytokines production [66]. Adipocyte death due to hypertrophy causes an inflammatory state [44,69]. The mononuclear cells of adipose tissue produce pro-inflammatory cytokines [6,44,81]. Excess abdominal fat is also responsible for the inflammatory condition [6,44,81]. 

Obesity also plays a role in hyperinsulinemia, IR, and HA occurrence. Visceral obesity arouses an increase in non-esterified fatty acids (NEFAs) levels in the blood. Skeletal muscles uptake NEFAs as the energy source instead of glucose. This hyperglycemia leads to a pancreas rapid reaction and hyperinsulinemia [55]. In addition, the lipolytic response of visceral fat to catecholamines causes lipotoxicity [44] and impairment of insulin clearance and activity [81]. 

FFA stimulates IRS-1 serine/threonine phosphorylation and reduces tyrosine phosphorylation. Increased FFAs reduce insulin and glucose uptake sensitivity in intramyocellular lipids [52]. Notably, that visceral fat is weightier in IR than abdominal [44] and subcutaneous fat [81] as the visceral fat lipolytic response to catecholamines is more severe [44,81]. The reason is the increased function of the β3 and higher expression of β1 and β2 receptors [81]. Moreover, the type 1 isoenzyme of 11β-hydroxysteroid dehydrogenase (11β-HSD) is involved in converting cortisone to active cortisol, which is highly expressed in adipose tissue, especially in adipose tissue visceral ones. Glucocorticoids reduce glucose uptake and insulin signaling in omental adipocytes [81]. In addition, visceral fat’s adiponectin secretion is less than subcutaneous fats, and this phenomenon leads to decreased adiponectin secretion in obesity [46]. 

In addition to all adipose tissue’s functions mentioned above, this tissue has endocrine function and secretes chemicals called adipokines or adipocytokines. Adipocytes produce leptin, a high concentration of which inhibits the expression of aromatase mRNA in granulosa cells—thus interrupting androgens to estrogen conversion [52]. In addition, it is suggested that increased leptin levels are related to the absence of folliculogenesis [81]. Moreover, adiponectin, secreted by adipocytes [52], has insulin-sensitizing, anti-diabetic, and anti-inflammatory effects [46]. The adiponectin insulin-sensitizing effect causes a reduction in FFA uptake and gluconeogenesis. It also plays a role in progesterone and estrogen production, ovulation, and decreased GnRH secretion [52]. Furthermore, adiponectin reduces LH secretion from the pituitary, triggers estradiol secretion in granulosa, and is associated with androgen production in ovaries [81]. Omentin-1, another adipose tissue secreted chemical, improves IGF-1-induced progesterone and estradiol secretion in different ways, including increasing the steroidogenic acute regulatory protein and CYP450 aromatase expression and enhancing IGF-1 receptor signaling [82].

Adipose tissue also has several enzymes responsible for converting androstenedione to testosterone and testosterone to dihydrotestosterone [45]. 17β-HSD converts androstenedione to testosterone [44,81] and estrone to estradiol [81]. This enzyme is expressed in adipose tissue [44,81]. As a result of this process, excess adiposity exacerbates HA [45].

Furthermore, the accumulation of lipid in non-adipose tissues, called lipotoxicity, causes oxidative/endoplasmic reticulum stress linked with inflammation and IR. Excess fatty acids in muscles and liver induce IR via serine phosphorylation of insulin receptor by diacylglycerol [83]. In addition, lipid accumulation in the liver diminishes HNF-4α levels leading to reduced SHBG production [63]. 

**Figure 1:**
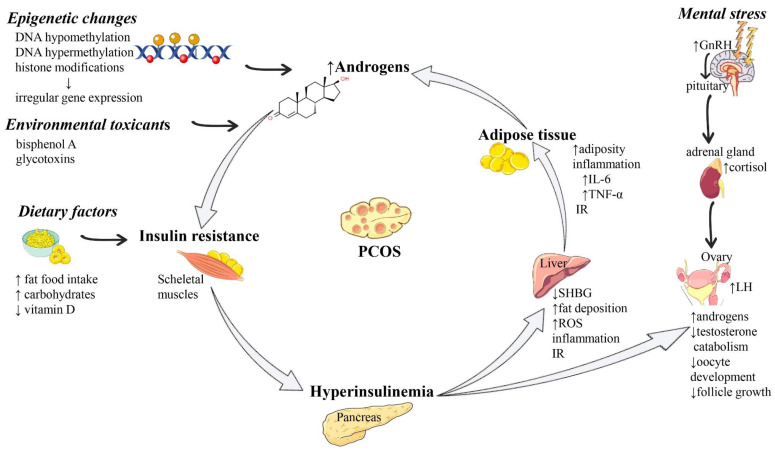
Summarized scheme regarding the pathophysiology of PCOS. Abbreviations and symbols: ↑ (increased), ↓ (decreased), DNA (deoxyribonucleic acid), GnRH (Gonadotropin-releasing hormone), IL-6 (interleukin 6), IR (insulin resistance), LH (luteinizing hormone), PCOS (polycystic ovary syndrome), SHBG (sex hormone-binding globulin), TNF-α (tumor necrosis alpha).

A summary of the most representative molecular mechanisms of PCOS pathogenesis is presented in Figure 1.

## 5. Management

The management approach and selection of the best therapy option depend on the target patient and her priorities [4]. The complications may vary from seeking fertility, regulation of menstrual disturbances to weight reduction or relief from hyperandrogenic symptoms, including acne, hirsutism, or androgenic alopecia [84]. Indeed, the approach should be individualized for each person to meet the optimal result [8]. There is no one ideal treatment for all women diagnosed with PCOS, which leaves physicians no choice but symptomatic therapy [85].

### 5.1. Lifestyle Modification and Non-Pharmacological Approaches

#### 5.1.1. Weight Loss

Elevated androgenic hormone levels lead to weight gain in women with PCOS, mainly in the abdominal area. As a result, many PCOS women have an apple shape body instead of a pear shape [37]. The first step for women diagnosed with PCOS would be weight reduction and calorie intake restriction [86]. Many studies demonstrate that even a 5% to 10% reduction in weight can restore the regular menstruation cycle [87]. For obese women, it would be best if they could reach their normal range of body mass index (BMI). Along with weight loss, the level of free testosterone decreases, and the incidence of metabolic syndrome reduces [84].

#### 5.1.2. Diet

As mentioned above, to achieve specific goals for each woman, the best diet or nutrient regimen would be the tailored one [87]. However, some suggestions may help choose what to eat more or less. An ideal diet would be rich in fibers and low in saturated fats and carbohydrates. There is a carbohydrate classification considering the blood glucose response they cause within 2 h: low and high glycemic index carbohydrates. Low glycemic index carbohydrates are at the top of our agenda; they include foods and vegetables like broccoli, raw carrot, lentils, soy, bran breakfast cereals, whole-grain bread, etc. Patients should also be aware that foods with a high glycemic index for prevention, white rice, cakes and cookies, fries or chips, and some fruits such as pineapple or watermelon are actual examples [37]. 

#### 5.1.3. Exercise

Exercise and physical activity play a key role in weight reduction. They may be beneficial to improve insulin sensitivity [88]. Different studies suggest various times for exercise during the week, but the American Heart Association (AHA) recommends approximately 150 min of moderate or 75 min of vigorous and intense exercise per week [84]. Several studies show that exercise, with or without being on a diet, can resume ovulation in women with PCOS. Exercise probably can affect ovulation through modulation of the hypothalamic-pituitary-gonadal (HPG) axis. In overweight and obese women, exercise leads to lower insulin and free androgen levels, inducing the restoration of HPA regulation of ovulation [88].

### 5.2. Complementary and Alternative Medicine (CAM)

Current management and accessible medications are only moderately effective in PCOS, and there are still some cases left untreated despite non-pharmacological and pharmacological treatments. Some literature claims that pharmacologically based therapies are only effective in 60% of patients [64]. Recent studies have demonstrated that using complementary and alternative medicine (CAM) as adjunctive therapy may benefit the management [89]. Today, CAM is a well-known approach that has been used at least at one point in more than 70% of PCOS patients during their diseases [90]. Several manners can categorize it; according to the latest edition of the National Center for Complementary and Integrative Health (NCCIH), complementary approaches can be classified by their primary therapeutic input into three classes of nutritional, psychological, physical, or all of them in combination [68]. One of the significant merits of CAM is that people often tend to accept these methods due to their beliefs and cultures; this leads to their improved adherence or tolerance to the therapy. Taking a look at prior studies, various methods of CAM including traditional Chinese medicine (TCM), immunotherapy, diet therapy (herbal and medicinal foods, probiotics, and vitamin or supplementation therapy), psychotherapy, spa, yoga, Tai Chi, and oxygen therapy have been considered as effective strategies in reducing the severity of PCOS and its complications [89,91,92,93,94,95]. Two critical subgroups of CAM effective in PCOS management are discussed in the following sections. 

#### 5.2.1. Acupuncture 

Acupuncture, a fundamental part of CAM, has been used in China for more than 3000 years [89]. It is a kind of sensory stimulation in which thin needles are placed into the skin and muscles. Acupuncture improves clinical manifestations of PCOS by activating somatic afferent nerves of the skin and muscles, modulating somatic and autonomic nervous system activity and endocrine/metabolic functions [91]. Within acupuncture, β-endorphin production increases, affecting the secretion of gonadotropin-releasing hormone, ovulation, and menstrual cycle. This means that acupuncture may induce ovulation and restore the menstrual cycle [64]. 

#### 5.2.2. Supplementations

Apart from medications with USFDA approval, plenty of supplementation products has been shown to be effective in some women with PCOS. These products include vitamin D supplements, resveratrol, α-lipoic acid, omega-3, berberine, folic acid, myoinositol (MI), and d-chiro-inositol (DCI). 

Vitamin D is effective in several studies, especially in cold seasons of the year. The deficiency of this vitamin is thought to be important in the pathogenesis of PCOS, so just the compensatory amount would be suggested [96]. 

Resveratrol is among the most recommended supplements for the treatment of PCOS. It is assumed to possess chemopreventive, anti-inflammatory and antioxidant, cardioprotective, and neuroprotective effects [97]. Resveratrol may play a beneficial role in PCOS by inhibiting HMG-CoA reductase expression and activity, just like statins [98]. Clinical use of this product has been shown to reduce IR and the risk of type 2 diabetes development [99]. 

Alpha-lipoic acid and omega-3 are the two supplements found to improve women’s lipid profile and insulin sensitivity through their anti-inflammatory and antioxidant properties [4]. 

Berberine is a nutraceutical compound with possible, desirable effects against IR and obesity, particularly against visceral adipose tissue (VAT) [100]. Folic acid is usually an agent given to PCOS women seeking fertility [24]. 

Last but not least, MI and DCI are other essential and well-studied supplements for PCOS treatment. MI has been demonstrated to improve the activity of insulin receptors and can potentially restore the ovulatory function in most women with PCOS [85]. Inositol influences intracellular metabolic processes; it activates key enzymes controlling glucose’s oxidative and nonoxidative metabolism. Studies conducted on PCOS women taken MI alone, DCI alone, and these combinations of the two showed that they cause increased frequency of ovulation, decreased need for FSH therapy for triggering the ovulation, and a significant improvement in the pregnancy rate [97].

### 5.3. Pharmacological Treatments

Before heading to pharmacological approaches, healthy lifestyle advice must be given to all women diagnosed with PCOS regardless of their weight, complaint, or anything else. This is because, in most cases, and especially in mild to moderate forms, women can solely benefit from diet and exercise [101]. However, the treatment would rely mainly on the patient’s choices and condition in others. If the patient does not want to get pregnant and complains mostly about her menstruation irregularity, combined oral contraceptives (COCs) or progestins are the drugs of choice. The physician can choose the best oral contraceptive with a look on other symptoms rather than menstruation irregularity; for example, Yasmin^®^, Yaz^®^, or some other agents can show antiandrogenic effects and can, on the other hand, result in the reduction of androgen production. As a result, they might be helpful in those with hirsutism and/or acne complications.

Metformin, from the biguanides category, is usually prescribed along with the first-choice drugs (COCs) to restore the ovulation cycle in PCOS women because of its insulin sensitivity-increasing properties. Metformin has an antihyperandrogenic effect in the short term too.

In other patients who just want relief from dermatological manifestations due to hyperandrogenism, agents such as aldosterone receptor antagonists (e.g., spironolactone) and 5-alpha reductases (e.g., finasteride) would be more beneficial. Therapy options change for those with infertility who should take agents for ovulation induction like clomiphene citrate and/or aromatase inhibitors [84]. 

Of course, there are lots of limitations and precautions, and not everyone can benefit from the agents mentioned above owing to their adverse effects or contraindications. Many COC agents cause nausea and vomiting as they try to stimulate the pregnancy situation for the body. In addition, depression, headaches, and migraine are commonly seen in those taking them. Metformin also causes nausea and vomiting in the first days of consumption which may not be tolerated in all patients and leads to abandonment of the therapy. Spironolactone, a widely used and prescribed agent for androgen-related complications, can cause hyperkalemia. Therefore, it is suggested to look up the adverse reactions or contraindications in reliable drug literature or ask the patient’s history of any possible reaction before the prescription.

The complete list of the routine medications used to treat PCOS and the step-by-step treatment pathway considering the patient’s complaints are provided here in Table 1.

### 5.4. Drug Repurposing in PCOS

Drug repurposing, or in other terms drug repositioning or drug re-tasking, actually means finding new indications in other diseases or conditions for a medication that has previously been in the market and has USFDA approval for a specific therapeutic goal [9]. Using this method has shortened the duration of the research and development process, given the thought that the medicines have passed pre-clinical and clinical, safety, and immunological tests. As mentioned before in this review, PCOS still does not have a single ideal pharmacological treatment, and doctors typically tend to cure patients’ symptoms with other agents. Taking a look at other drugs—mostly diabetes agents—may be helpful to recognize some new medications for women with PCOS-related complications. 

Table 2 and Table 3, respectively, present general information and clinical trials of drugs primarily approved for other indications (e.g., diabetes type II, hyperlipidemia, weight reduction, etc.), which are now being examined to see their potential effect in PCOS. 

## 6. Conclusions

Although the pathogenesis of PCOS is not fully understood, it is believed that different factors from epigenetic alterations to obesity, inflammation, and inactivity may aggravate this syndrome. Since there is still no certain medication or definite cure for this condition, the routine approach after advising on some lifestyle modification and supplementary tips is symptomatic therapy with plenty of agents, including contraceptives, oral antidiabetics, or antiandrogens. In terms of the repurposing, there is a good chance that other approved agents could exert beneficial effects on PCOS. Since the complete profiles of these agents are available, and their efficacy and safety have already been comprehensively studied, the pathway for finding novel treatments becomes a little more straightforward. However, there is still very much to discover and examine for a better understanding of the pathogenesis, and, as a result, targeting the mechanism by proper medication.

## Figures and Tables

**Table 1:** Commonly prescribed medications in PCOS.

Generic Name (Brand)/Ref	Mechanism of Action	Purpose of Therapy	Common Side Effects	Contraindications
COCs (estrogen and progestin)				
Levonorgestrel/Ethinyl estradiol—LD and HD [100]	Inhibition of ovulation via negative feedback on the hypothalamus alters the regular pattern of gonadotropin secretion of FSH and LH from the anterior pituitary gland	Menstrual cyclicity, hirsutism, acne	Nausea and vomiting (especially at first), breast tenderness, breakthrough bleeding, weight gain, acne or darkening of facial skin, etc.	Oral contraceptives should not be used in women who currently have the following conditions: Thrombophlebitis or thromboembolic disorders A history of deep vein thrombophlebitis or thromboembolic disorders Cerebral vascular or coronary artery disease Known or suspected carcinoma of the breast Carcinoma of the endometrium or other known or suspected estrogen-dependent neoplasia Undiagnosed abnormal genital bleeding Cholestatic jaundice of pregnancy or jaundice with prior pill use Hepatic adenomas or carcinomas Known or suspected pregnancy
Desogestrel/Ethinyl estradiol (Marvelon^®^, Mircette^®^) [101]		Menstrual cyclicity	Depression, headache, migraine, mood changes, skin rash, urticaria, decreased or increased libido, weight gain or loss, abdominal pain, diarrhea, nausea and vomiting, breast hypertrophy and tenderness, vaginal discharge, hypersensitivity reactions, etc.	
Cyproterone acetate/Ethinyl estradiol (Diane 35^®^) [102]		Menstrual cyclicity	Dysmenorrhea, breast tenderness, change in libido, headache, depression, nervousness, chloasma, varicosity, edema, dizziness	
Drospirenone/Ethinyl estradiol (Yasmin^®^) [103]		Menstrual cyclicity, hirsutism, acne	PMS, headache or migraine, breast pain/tenderness/discomfort, nausea and vomiting, abdominal pain/tenderness/discomfort, mood changes	
Drospirenone/Ethinyl estradiol (Yaz^®^) [104]		Menstrual cyclicity, hirsutism, acne	Headache or migraine, menstrual irregularities, nausea and vomiting, breast pain or tenderness, mood changes, fatigue, irritability, decreased libido, increased weight	
Dienogest/estradiol valerate (Natazia^®^) [105]		Menstrual cyclicity, hirsutism, acne	Headaches, irregular uterine bleeding, breast tenderness, nausea and vomiting, acne, and increased weight	
Chlormadinone acetate/Ethinyl estradiol (Belara^®^) [106]		Menstrual cyclicity, hirsutism, acne	Breast pain or tension, depressed state, loss of libido, migraine or headache	
Progestins				
Medroxyprogesterone acetate (Provera^®^) [107]	Inhibition of secretion of pituitary gonadotropins, Prevention of follicular maturation and ovulation	Menstrual cyclicity,	Amenorrhea, change in menstrual flow, hot flash, weight gain or weight loss, menstrual disease, abdominal pain, headache, nervousness	Undiagnosed abnormal genital bleeding Known, suspected, or history of cancer of the breast Known or suspected estrogen, progesterone dependent neoplasia Active deep vein thrombosis, pulmonary embolism, or a history of these conditions Active or recent arterial thromboembolic disease (e.g., stroke and myocardial infarction) Known liver dysfunction or disease Missed abortion As a diagnostic test for pregnancy Known hypersensitivity to the tablets Known or suspected pregnancy
Biguanides				
Metformin (Glucophage^®^) [108]	↓hepatic glucose production ↓intestinal absorption, ↑insulin sensitivity	Impaired glucose tolerance, type II diabetes	Diarrhea, nausea and vomiting, flatulence, asthenia, indigestion, abdominal discomfort, headache	Severe renal impairment Known hypersensitivity to metformin hydrochloride Acute or chronic metabolic acidosis, including diabetic ketoacidosis, with or without coma
Antiandrogens				
Spironolactone (Aldactone^®^) [109]	Competitive antagonistic activity against aldosterone receptors causes potassium retention, sodium and water excretion	Hirsutism, acne	Gynecomastia	Hyperkalemia Addison’s disease Concomitant use of eplerenone
Finasteride (Propecia^®^) [110]	A competitive and specific inhibitor of Type II 5-alpha-reductase, an enzyme that converts the androgen testosterone into DHT	Hirsutism, acne	Decreased libido, erectile dysfunction, and ejaculation disorder	Pregnancy Hypersensitivity to any components of this product
Antiestrogens				
Clomiphene citrate (Clomid^®^) [111]	Occupying ERs for a longer duration than estrogen, inhibiting normal estrogen negative feedback, which results in increased pulsatile GnRH secretion from the hypothalamus and pituitary gonadotropin release	Ovulation induction	Ovarian enlargement, vasomotor flushes, abdominal-pelvic discomfort/distention/bloating, nausea and vomiting, breast discomfort, blurred vision, lights/ floaters/waves/unspecified visual complaints, photophobia, diplopia, scotomata, phosphenes, headache, abnormal uterine bleeding, intermenstrual spotting, menorrhagia	Hypersensitivity to any components of this product Pregnancy Liver disease or a history of liver dysfunction Abnormal uterine bleeding of undetermined origin Ovarian cysts or enlargement not due to PCOS Uncontrolled thyroid or adrenal dysfunction In the presence of an organic intracranial lesion such as pituitary tumor
Aromatase inhibitors				
Letrozole (Femara^®^) [112]	Nonsteroidal competitive inhibitor of the aromatase enzyme which catalyzes the conversion of androgens to estrogens	Ovulation induction	Hot flashes, arthralgia, flushing, asthenia, edema, arthralgia, headache, dizziness, hypercholesterolemia, sweating increased, bone pain	Women of premenopausal endocrine status, including pregnant women

Abbreviations: COC: Combined oral contraceptives; DHT: Dihydrotestosterone; ER: Estrogen receptor; FSH: Follicle-stimulating hormone; GnRH: Gonadotropin-releasing hormone; HD: High dose; IM: Intramuscular; LD: Low dose; LH: Luteinizing hormone; PCOS: Polycystic ovary syndrome; PMS: Premenstrual syndrome; Ref: Reference; XR: Extended-release.

**Table 2:** Repurposed medications for the treatment of PCOS.

Generic Name (Brand)/Ref	Pharmacological Category	Mechanisms of Action	USFDA Approved Indication(s)	Common Adverse Events (≥5%)	Contraindications and Drug Interactions
Simvastatin (Zocor^®^) [113]	Antilipemic HMG-CoA reductase inhibitor	↓ HMG-CoA ↓ cholesterol biosynthesis	Heterozygous familial hypercholesterolemia, homozygous familial hypercholesterolemia, prevention of atherosclerotic cardiovascular disease	Upper respiratory infections headache abdominal pain constipation nausea	Hypersensitivity to any component of this medication Concomitant administration of potent CYP3A4 inhibitors Concomitant administration of gemfibrozil, cyclosporine, or danazol Active liver disease, which may include unexplained persistent elevations in hepatic transaminase levels Women who are pregnant or may become pregnant and nursing mothers
Atorvastatin (Lipitor^®^) [114]				Nasopharyngitis arthralgia diarrhea pain in extremity	Hypersensitivity to atorvastatin Active liver disease, unexplained persistent elevation of serum transaminases Pregnancy, breastfeeding Concurrent therapy with cyclosporine, clarithromycin, itraconazole, HIV protease inhibitors (ritonavir plus saquinavir or lopinavir plus ritonavir), etc.
Pioglitazone (Actos^®^) [115]	Antidiabetic thiazolidinedione	↓ blood glucose ↑ target cell response to insulin	Treatment of type II diabetes mellitus	Upper respiratory tract infection, headache, pharyngitis, sinusitis myalgia,	Do not initiate in patients with established NYHA Class III or IV heart failure Do not use in patients with a history of a severe hypersensitivity reaction to it
Empagliflozin (Jardiance^®^) [116]	Antidiabetic SGLT-2 inhibitor	↓ reabsorption of filtered glucose from the tubular lumen of the proximal renal tubules lowering the renal threshold for glucose	Treatment of type II diabetes mellitus	Urinary tract infections female genital mycotic infections	History of severe hypersensitivity reaction Severe renal impairment, end-stage renal disease, or dialysis
Dapagliflozin (Farxiga^®^) [117]			Treatment of type II diabetes mellitus, heart failure with a reduced ejection fraction	Female genital mycotic infections, nasopharyngitis, urinary tract infections	History of severe hypersensitivity reaction Severe renal impairment, end-stage renal disease, or dialysis
Canagliflozin (Invokana^®^) [118]			Treatment of type II diabetes mellitus	Female genital mycotic infections, urinary tract infection, increased urination	History of serious hypersensitivity reaction Severe renal impairment, ESRD, or on dialysis
Sitagliptin (Januvia^®^) [119]	Antidiabetic DPP-4 inhibitor	↓ DPP-4 enzyme, ↑ incretin levels, ↑ insulin synthesis by pancreatic beta cells.	Treatment of type II diabetes mellitus	Upper respiratory tract infection, nasopharyngitis, headache	History of a severe hypersensitivity reaction, such as anaphylaxis or angioedema
Liraglutide (Saxenda^®^, Victoza^®^) [120]	Antidiabetic GLP-1 receptor agonist	Long-acting analog of GLP-1 ↑ glucose-dependent insulin secretion ↓ gastric emptying ↓ food intake ↓ inappropriate glucagon secretion	Treatment of type II diabetes mellitus, weight management	↑ heart rate, hypoglycemia, constipation/diarrhea, nausea and vomiting, gastroenteritis, ↓ appetite, ↓ dyspepsia	In patients with a personal or family history of MTC or patients with MEN-2 History of a severe hypersensitivity reaction to the drug or any component of the formulation
Semaglutide (Ozempic^®^) [30]			Treatment of type II diabetes mellitus	Abdominal pain, nausea, vomiting, constipation, diarrhea	
Exenatide (Byetta^®^) [121]			Treatment of type II diabetes mellitus	Nausea and vomiting, hypoglycemia, diarrhea, feeling jittery, dizziness, headache, dyspepsia	History of a severe hypersensitivity reaction to the drug or any component of the formulation
N-acetyl cysteine (Cetylev^®^) [122]	Antidote, mucolytic agent	↑ glutathione, mucolytic	Mucolytic, acetaminophen overdose	Nausea and vomiting, autoimmune disease, anaphylactoid reaction	History of a severe hypersensitivity reaction to the drug or any component of the formulation Hypersensitivity reactions, including urticarial ↑ Risk of upper GI bleeding

Abbreviations: CYP3A4: Cytochrome P450 3A4; DPP-4: Dipeptidyl peptidase 4; GI: Gastrointestinal; GLP-1: Glucose-like peptide 1; HIV: Human immunodeficiency virus; HMG-CoA: β-Hydroxy-β-methylglutaryl-CoA; MEN-2: Multiple endocrine neoplasia syndrome type 2; MTC: Medullary thyroid carcinoma; NYHA: New York Heart Association; Ref: Reference; SGLT-2: Sodium-glucose cotransporter-2; USFDA: United States Food and Drug Administration.

**Table 3:** Clinical trials of the repurposed medications for PCOS since 2016.

Treatment (Tx) Phase/Year First Posted Ref	Dosage (Duration of Therapy)	Subjects Condition Groups	Study Design	Results	Non-Serious AEs (%) (Treatment-Related)	Serious AEs (%) (Treatment-Related)
Astaxanthin Not applicable/2019 [123]	Exp: 8 mg astaxanthin + clomiphene citrate P: matching placebo + clomiphene citrate (NA)	48 PCOS Exp = NA P = NA	R, PA, QB	NA	NA	NA
Berberine Not applicable/2021 [124]	550 mg berberine tablet, BID (before lunch and dinner) (NA)	12 PCOS	SGA, OL	NA	NA	NA
Dapagliflozin and exenatide Phase III/2019 [125]	Exp 1: 2 mg exenatide, SC injection, once every week Exp 2: 10 mg dapagliflozin pill, PO, QD, a.m. Exp 3: 2 mg exenatide, SC injection, once every week + 10 mg dapagliflozin pill, PO, QD, a.m. Exp 4: 5 mg dapagliflozin-1000 mg glucophage pill, PO, QD, a.m. for 4 weeks after those 2 pills, PO, QD, a.m. AC: 3.75 mg phentermine-23 mg topiramate pill, PO, QD, a.m. for 2 weeks after that 7.5 mg pheniramine-46 mg topiramate pill, PO, QD, a.m. (24 weeks)	119 PCOS, obesity Exp1 = 23 Exp2 = 23 Exp3 = 22 Exp4 = 26 AC = 25	R, PA, SB	NA	Yeast infection or UTI, kidney stone, nausea and upset stomach, insomnia, fatigue, headache, lightheadedness, injection site reaction, vaginal irritation, prolonged menstrual cycle, rapid heartbeat, stuffy nose	Pregnancy
D-chiro-inositol and myo-inositol Phase II and phase III/2017 [126]	Exp: 500 mg myo-inositol capsule, BID (every 12 h) + 150 mg D-chiro-inositol capsule, BID (every 12 h) AC: 500 mg myo-inositol capsule, BID (every 12 h) + 13.8 mg D-chiro-inositol capsule, BID (every 12 h) (12 weeks)	60 PCOS, infertility Exp = NA AC = NA	R, PA, QB, controlled, multicenter	NA	NA	NA
Empagliflozin Phase II and phase III/2017 [127,128]	Exp: 25 mg empagliflozin per day AC: 1500 mg metformin per day (3 months)	40 PCOS, irregular period and biochemical hyperandrogenism Exp = 19 C = 20	R, PA, OL	Significant improvement in anthropometric parameters and body composition in overweight or obese women in the empagliflozin group, no change in hormonal or metabolic parameters	Headache dizziness, mild rash	Not reported
Exenatide Phase IV/2017 [129,130]	Exp1: 5 mcg or 10 mcg exenatide, BID AC: 500 mg metformin, TID or 1000 mg metformin, BID Exp2: 5 mcg or 10 mcg exenatide, BID + 500 mg metformin, TID or 1000 mg metformin, BID (12 weeks)	183 PCOS, overweight or obesity, a disorder of glucose regulation Exp1 = 61 AC = 61 Exp2 = 61	R, PA, OL	Compared with metformin monotherapy, exenatide alone or in combination with metformin gives a higher remission rate in pre-diabetes PCOS women as it improves postprandial insulin secretion.	Nausea and vomiting, headache	Not reported
Folic acid and myo-inositol Not applicable/2022 [131]	Exp: 200 mcg folic acid, 2 g myo-inositol, 50 mg alpha-lactalbumin, BID (6 months)	36 PCOS, insulin resistance Exp = 36	R, SGA, OL	NA	NA	NA
Folic acid and myo-inositol Not applicable/2018 [132,133]	Exp1: 2 mg myo-inositol, 0.2 mg folic acid, PO, BID Exp2: 2 mg myo-inositol, 0.2 mg folic acid, 50 mg alpha-lactalbumin, PO, BID (3 months)	37 PCOS, anovulation, and infertility more than one year Exp1 = 37 Exp2 = 14	NR, SA, OL	Administration of myo-inositol with alpha-lactalbumin improves PCOS treatment in a patient resistant to myo-inositol	NA	NA
Folic acid and myo-inositol Not applicable/2018 [134]	2000 mg myo-inositol-500 mg L-tyrosine-40 mcg chromium picolinate-55 mcg selenium-200 mcg folic acid sachet, QD (6 months)	186 PCOS, menstrual problems, hirsutism	SGA, OL	Improve in symptoms	NA	NA
Folic acid and omega-3 free fatty acid Not applicable/2017 [135,136]	Exp: omega-3 fatty acid, 500 mg soft capsule, QD + 800 mg folic acid-70 mg selenium- 30 mg vitamin E-4 mg catechin-12 mg glycyrrhizin-30 mg Q10 coenzyme tablet, QD P: folic acid, 200 mcg capsule, BID (3 months)	60 PCOS, infertility, micronutrient deficiency, oligomenorrhea, or complete amenorrhea for at least 90 days Exp = NA P = NA	R, PA, DB	LH/FSH ratio, testosterone, and AMH significantly decreased in the experimental group	NA	NA
Folic acid, myo-inositol, and liraglutide Phase IV/2018 [137]	AC1: dietary advice + 2 g of myo-inositol and folic acid, BID AC2: dietary advice + liraglutide starting at 0.6 mg, gradually increasing up to a dose of 3 mg per day after four weeks, pen injector (16 weeks)	21 PCOS, obesity, metabolic syndrome AC1 = NA AC2 = NA	R, PA, OL	NA	NA	NA
L-carnitine Not applicable/2018 [138]	Exp: 50 mg clomiphene citrate tablet, PO, BID, from the third day of the cycle until the seventh day of the cycle + 1 g carnitine tablet, PO, TID, from the third day of the cycle until the day of the pregnancy test AC: 50 mg clomiphene citrate tablet, PO, BID, from the third day of the cycle until the seventh day of the cycle (14 days)	106 PCOS Exp = 53 AC = 53	R, PA, DB	NA	NA	NA
L-carnitine Phase IV/2018 [139]	Exp: 50 mg clomiphene citrate tablet, PO, BID, from the third day of the cycle until the seventh day of the cycle + 1 g carnitine tablet, PO, TID, from the third day until the day of the pregnancy test AC: 50 mg clomiphene citrate tablet, PO, BID, from the third day of the cycle until the seventh day of the cycle + placebo tablet, PO, TID, from the third day until the day of the pregnancy test (NA)	150 PCOS, primary or secondary infertility Exp = NA AC = NA	R, PA, QB	NA	NA	NA
Liraglutide Phase III/2018 [140]	Exp: 0.6 mg liraglutide, SC pen injector, QD for one week, step up to 1.2 mg, SC pen injector, QD for one week, to 1.8 mg, SC pen injector, QD for one week, 2.4 mg, SC pen injector, QD for one week, to 3 mg, SC pen injector, QD final dose P: 0.6 mg placebo liraglutide, SC pen injector, QD for one week, step up to 1.2 mg, SC pen injector, QD for one week, to 1.8 mg, SC pen injector, QD for one week, 2.4 mg, SC pen injector, QD for one week, to 3 mg, SC pen injector (30 weeks)	88 PCOS, pre-diabetes, obesity android Exp = NA P = NA	R, PA, QB, PC	NA	NA	NA
N-acetyl cysteine Phase II and phase III/2016 [141]	AC1: 1200 mg N-acetyl cysteine per day for 5 days, starting from the second day of the cycle to the sixth day of the cycle + 50 mg clomiphene citrate, PO, BID + laparoscopic ovarian drilling AC2: 50 mg clomiphene citrate, PO, BID + laparoscopic ovarian drilling (NA)	144 PCOS, clomiphene citrate resistance, BMI between 25 and 30 kg/m^2^ AC1 = 60 AC2 = 60	R, PA, QB, PC	NA	NA	NA
Pioglitazone Early phase I/2016 [142]	AC1: 2 mg cyproterone acetate-35 mcg ethinyl estradiol tablet, QD + 50 mg spironolactone, BID AC2: 500 mg metformin, TID AC3: 30 mg pioglitazone, QD (3 months)	90 PCOS AC1 = NA AC2 = NA AC3 = NA	R, PA, TB	NA	NA	NA
Pioglitazone Early Phase I/2017 [143]	AC: 1000 mg metformin-30 mg pioglitazone tablet Exp: 500 mg metformin tablet, PO, BID (3 months)	106 PCOS AC = 53 Exp = 53	R, PA, DB	NA	Not reported	Not reported
Pioglitazone Early Phase I/2018 [144]	AC: 30 mg pioglitazone tablet, QD + 50 mg clomiphene citrate tablet, QD or BID (every 12 h), from the third day of menstrual cycle and continue for five days during treatment P: metformin 1500 mg, TID + clomiphene citrate 50 mg tablet, QD or BID (every 12 h), from the third day of menstrual cycle and continue for five days during treatment (3 months)	400 PCOS and infertility AC = NA P = NA	R, SGA, SB	NA	NA	NA
Resveratrol and myo-inositol Phase II/2021 [145]	AC: 1000 mg resveratrol, BID + 1000 mg myo-inositol, BID Standard therapy group: 500 mg metformin, BID + 15 mg pioglitazone, BID (NA)	88 PCOS, hirsutism or hyperandrogenism, oligo-ovulation or anovulation AC = 51 Standard therapy group = 51	R, PA, TB	NA	NA	NA
Sitagliptin Phase IV/2014 [146,147]	Exp1: 100 mg sitagliptin per day, PO, for 30 days, then one placebo pill per day, PO, for 30 days Exp2: one placebo pill per day, PO, for 30 days, then 100 mg sitagliptin per day, PO, for 30 days (60 days)	23 PCOS, BMI ≥ 30 kg/m^2^ Exp1 = 11 Exp2 = 12	R, CA, TB	↓Maximum glucose response to oral glucose tolerance test, ↓visceral adiposity, ↑growth hormone half-life, and interval pulse	Dizziness, headache, abdominal pain, nausea	Pancreatitis
Sitagliptin Phase IV/2017 [148]	Exp1: 100 mg sitagliptin per day + lifestyle intervention Exp2: lifestyle intervention (12 weeks)	30 PCOS, BMI ≥ 30 kg/m^2^ Exp1 = NA Exp2 = NA	R, PA, OL	NA	NA	NA
Vitamin D Phase III/2019 [149,150]	Exp: 6000 IU vitamin D per day, PO + 1000 mg calcium carbonate per day, PO + 1500 mg metformin per day, PO, starting with 500 mg QD for the first week, 500 mg BID in the second week, and 500 mg TID from the third week P: 1500 mg metformin per day, PO, starting with 500 mg QD for the first week, 500 mg BID in the second week, and 500 mg TID from the third week + placebo, PO (8 weeks)	40 PCOS, Vitamin D deficiency/insufficiency Exp = 20 P = 20	R, PA. SB, PC	Calcium and vitamin D may support metformin effect on menstrual cycle irregularities in PCOS patients suffering vitamin D deficiency/insufficiency	Headache, gastrointestinal side effects	Not reported
Vitamin D Not applicable/2019 [151]	AC: 42000 IU vitamin D per week, PO + 500 mg calcium carbonate per day P: 42000 IU vitamin D per week, PO + 500 mg calcium carbonate per day (12 weeks)	145 PCOS = 95 Healthy = 50 AC = 55 P = 90	R, PA, DB, PC	NA	NA	NA
Vitamin D Phase IV/2019 [152]	AC: 100,000 IU vitamin D per month, IM + 1000 mg metformin tablet, BID + 50 mg clomiphene citrate tablet, BID, from the second day to the sixth day of the cycle, starting from the third month to the fifth month P: 1000 mg metformin tablet, BID + 50 mg clomiphene citrate tablet, BID, from the second day to the sixth day of the cycle, starting from the third month to the fifth month (5 months)	120 PCOS, vitamin D deficiency, infertility, clomiphene resistance AC = 60 P = 60	R, PA, OL	NA	NA	NA

Abbreviations: a.m.: before noon; AC: active comparator; AEs: adverse effects; AMH: anti-mullerian hormone; BID: twice a day; BMI: body mass index; CA: crossover assignment; DB: double-blind; Exp: experimental; FSH: follicle stimulation hormone; g: gram; IM: intramuscular; IU: international unit; LH: luteinizing hormone; mcg: microgram; mg: milligram; NA: not available; OL: open-label; P: placebo; PA: parallel assignment; PC: placebo-controlled; PCOS: polycystic ovary syndrome; PO: per oral (by mouth); QB: quadruple blind; QD: once a day; R: randomized; Ref: reference; SB: single-blind; SC: subcutaneous; SGA: single group assignment; TB: triple blind; TID: three times a day; Tx: treatment; UTI: urinary tract infection.

## References

[1] Deans R. (2019). Polycystic ovary syndrome in adolescence. Med. Sci..

[2] Witchel S.F., E Oberfield S., Peña A.S. (2019). Polycystic Ovary Syndrome: Pathophysiology, Presentation, and Treatment With Emphasis on Adolescent Girls. J. Endocr. Soc..

[3] Polycystic Ovary Syndrome. https://www.womenshealth.gov/a-z-topics/polycystic-ovary-syndrome.

[4] Bednarska S., Siejka A. (2017). The pathogenesis and treatment of polycystic ovary syndrome: What’s new?. Adv. Clin. Exp. Med..

[5] Ganie M.A., Vasudevan V., Wani I.A., Baba M.S., Arif T., Rashid A. (2019). Epidemiology, pathogenesis, genetics & management of polycystic ovary syndrome in India. Indian J. Med Res..

[6] Glueck C.J., Goldenberg N. (2019). Characteristics of obesity in polycystic ovary syndrome: Etiology, treatment, and genetics. Metab..

[7] Damone A.L., Joham A.E., Loxton D., Earnest A., Teede H.J., Moran L.J. (2019). Depression, anxiety and perceived stress in women with and without PCOS: A community-based study. Psychol. Med..

[8] Escobar-Morreale H.F. (2018). Polycystic ovary syndrome: Definition, aetiology, diagnosis and treatment. Nat. Rev. Endocrinol..

[9] Sadeghi H.M., Adeli I., Mousavi T., Daniali M., Nikfar S., Abdollahi M. (2021). Drug Repurposing for the Management of Depression: Where Do We Stand Currently?. Life.

[10] Differential Diagnosis of PCOS. https://www.verywellhealth.com/what-is-the-differential-diagnosis-of-pcos-2616642.

[11] Witchel S.F., Burghard A.C., Tao R.H., Oberfield S.E. (2019). The diagnosis and treatment of PCOS in adolescents. Curr. Opin. Pediatr..

[12] Polycystic Ovary Syndrome (PCOS). https://www.mayoclinic.org/diseases-conditions/pcos/diagnosis-treatment/drc-20353443.

[13] Diagnosis of Polycystic Ovary Syndrome. https://www.nhs.uk/conditions/polycystic-ovary-syndrome-pcos/diagnosis/.

[14] European Society of Human Reproduction and Embryology (2018). International Evidence-Based Guideline for the Assessment and Management of Polycystic Ovary Syndrome. https://www.eshre.eu/Guidelines-and-Legal/Guidelines/Polycystic-Ovary-Syndrome.

[15] Ilie I.R., Georgescu C.E. (2015). Polycystic Ovary Syndrome-Epigenetic Mechanisms and Aberrant MicroRNA. Adv. Virus Res..

[16] Casadesús J., Noyer-Weidner M., Maloy S., Hughes K. (2013). Epigenetics. Brenner’s Encyclopedia of Genetics.

[17] Mukherjee S., Sagvekar P., Azarnezhad R., Patil K. (2018). Pathomechanisms of polycystic ovary syndrome Multidimensional approaches. Front. Biosci..

[18] Ibanez L., Oberfield S.E., Witchel S.F., Auchus R.J., Chang R.J., Codner E., Dabadghao P., Darendeliler F., Elbarbary N., Gambineri A. (2017). An International Consortium Update: Pathophysiology, Diagnosis, and Treatment of Polycystic Ovarian Syndrome in Adolescence. Horm. Res. Paediatr..

[19] Fenichel P., Rougier C., Hieronimus S., Chevalier N. (2017). Which origin for polycystic ovaries syndrome: Genetic, environmental or both?. Ann. d’Endocrinol..

[20] Abbott D.H., A Dumesic D., E Levine J. (2019). Hyperandrogenic origins of polycystic ovary syndrome – implications for pathophysiology and therapy. Expert Rev. Endocrinol. Metab..

[21] Rutkowska A., Diamanti-Kandarakis E. (2016). Polycystic ovary syndrome and environmental toxins. Fertil. Steril..

[22] Qu F., Wang F.-F., Yin R., Ding G.-L., El-Prince M., Gao Q., Shi B.-W., Pan H.-H., Huang Y.-T., Jin M. (2012). A molecular mechanism underlying ovarian dysfunction of polycystic ovary syndrome: Hyperandrogenism induces epigenetic alterations in the granulosa cells. J. Mol. Med..

[23] Li Y., Chen C., Ma Y., Xiao J., Luo G., Li Y., Wu D. (2019). Multi-system reproductive metabolic disorder: Significance for the pathogenesis and therapy of polycystic ovary syndrome (PCOS). Life Sci..

[24] Rocha A.L., Oliveira F.R., Azevedo R.C., Silva V.A., Peres T.M., Candido A.L., Gomes K.B., Reis F.M. (2019). Recent advances in the understanding and management of polycystic ovary syndrome. F1000Research.

[25] Jones L., Regan F., Worsfold P., Poole C., Townshend A., Miró M. (2019). Endocrine Disrupting Chemicals. Encyclopedia of Analytical Science.

[26] Merkin S.S., Phy J.L., Sites C.K., Yang D. (2016). Environmental determinants of polycystic ovary syndrome. Fertil. Steril..

[27] Calina D., Docea A.O., Golokhvast K.S., Sifakis S., Tsatsakis A., Makrigiannakis A. (2019). Management of Endocrinopathies in Pregnancy: A Review of Current Evidence. Int. J. Environ. Res. Public Health.

[28] Sobolewski M., Barrett E.S. (2014). Polycystic Ovary Syndrome: Do Endocrine-Disrupting Chemicals Play a Role?. Semin. Reprod. Med..

[29] Soave I., Occhiali T., Assorgi C., Marci R., Caserta D. (2020). Environmental toxin exposure in polycystic ovary syndrome women and possible ovarian neoplastic repercussion. Curr. Med Res. Opin..

[30] Palioura E., Diamanti-Kandarakis E. (2015). Polycystic ovary syndrome (PCOS) and endocrine disrupting chemicals (EDCs). Rev. Endocr. Metab. Disord..

[31] Palioura E., Diamanti-Kandarakis E. (2013). Industrial endocrine disruptors and polycystic ovary syndrome. J. Endocrinol. Investig..

[32] Wang J., Wu D., Guo H., Li M. (2019). Hyperandrogenemia and insulin resistance: The chief culprit of polycystic ovary syndrome. Life Sci..

[33] Stefanaki C., Pervanidou P., Boschiero D., Chrousos G.P. (2018). Chronic stress and body composition disorders: Implications for health and disease. Hormones.

[34] Steegers-Theunissen R., Wiegel R., Jansen P., Laven J., Sinclair K. (2020). Polycystic Ovary Syndrome: A Brain Disorder Characterized by Eating Problems Originating during Puberty and Adolescence. Int. J. Mol. Sci..

[35] Yang S., Yang C., Pei R., Li C., Li X., Huang X., Wu S., Liu D. (2018). Investigation on the association of occupational stress with risk of polycystic ovary syndrome and mediating effects of HOMA-IR. Gynecol. Endocrinol..

[36] Szczuko M., Kikut J., Szczuko U., Szydłowska I., Nawrocka-Rutkowska J., Ziętek M., Verbanac D., Saso L. (2021). Nutrition Strategy and Life Style in Polycystic Ovary Syndrome—Narrative Review. Nutrients.

[37] Faghfoori Z., Fazelian S., Shadnoush M., Goodarzi R. (2017). Nutritional management in women with polycystic ovary syndrome: A review study. Diabetes Metab. Syndr. Clin. Res. Rev..

[38] Muscogiuri G., Altieri B., de Angelis C., Palomba S., Pivonello R., Colao A., Orio F. (2017). Shedding new light on female fertility: The role of vitamin D. Rev. Endocr. Metab. Disord..

[39] Ciebiera M., Esfandyari S., Siblini H., Prince L., Elkafas H., Wojtyła C., Al-Hendy A., Ali M. (2021). Nutrition in Gynecological Diseases: Current Perspectives. Nutrients.

[40] Greenwood E.A., Huddleston H.G. (2019). Insulin resistance in polycystic ovary syndrome: Concept versus cutoff. Fertil. Steril..

[41] Petrakis D., Vassilopoulou L., Mamoulakis C., Psycharakis C., Anifantaki A., Sifakis S., Docea A.O., Tsiaoussis J., Makrigiannakis A., Tsatsakis A.M. (2017). Endocrine Disruptors Leading to Obesity and Related Diseases. Int. J. Environ. Res. Public Health.

[42] Shang Y., Zhou H., Hu M., Feng H. (2020). Effect of Diet on Insulin Resistance in Polycystic Ovary Syndrome. J. Clin. Endocrinol. Metab..

[43] Dabadghao P. (2019). Polycystic ovary syndrome in adolescents. Best Pr. Res. Clin. Endocrinol. Metab..

[44] Rosenfield R.L., Ehrmann D.A. (2016). The Pathogenesis of Polycystic Ovary Syndrome (PCOS): The Hypothesis of PCOS as Functional Ovarian Hyperandrogenism Revisited. Endocr. Rev..

[45] Rothenberg S.S., Beverley R., Barnard E., Baradaran-Shoraka M., Sanfilippo J.S. (2018). Polycystic ovary syndrome in adolescents. Best Pr. Res. Clin. Obstet. Gynaecol..

[46] Jeanes Y., Reeves S. (2017). Metabolic consequences of obesity and insulin resistance in polycystic ovary syndrome: Diagnostic and methodological challenges. Nutr. Res. Rev..

[47] Polak K., Czyzyk A., Simoncini T., Meczekalski B. (2017). New markers of insulin resistance in polycystic ovary syndrome. J. Endocrinol. Investig..

[48] Zhang C., Hu J., Wang W., Sun Y., Sun K. (2020). HMGB1-induced aberrant autophagy contributes to insulin resistance in granulosa cells in PCOS. FASEB J..

[49] He F.-F., Li Y.-M. (2020). Role of gut microbiota in the development of insulin resistance and the mechanism underlying polycystic ovary syndrome: A review. J. Ovarian Res..

[50] Bannigida D.M., Nayak B.S., Vijayaraghavan R. (2018). Insulin resistance and oxidative marker in women with PCOS. Arch. Physiol. Biochem..

[51] Avery P.J., Jorgensen A., Hamberg A.K., Wadelius M., Pirmohamed M., Kamali F. (2011). A Proposal for an Individualized Pharmacogenetics-Based Warfarin Initiation Dose Regimen for Patients Commencing Anticoagulation Therapy. Clin. Pharmacol. Ther..

[52] Zeng X., Xie Y.-J., Liu Y.-T., Long S.-L., Mo Z.-C. (2020). Polycystic ovarian syndrome: Correlation between hyperandrogenism, insulin resistance and obesity. Clin. Chim. Acta.

[53] Docea A.O., Vassilopoulou L., Fragou D., Arsene A.L., Fenga C., Kovatsi L., Petrakis D., Rakitskii V.N., Nosyrev A.E., Izotov B.N. (2017). CYP polymorphisms and pathological conditions related to chronic exposure to organochlorine pesticides. Toxicol. Rep..

[54] Cassar S., Misso M.L., Hopkins W.G., Shaw C.S., Teede H., Stepto N.K. (2016). Insulin resistance in polycystic ovary syndrome: A systematic review and meta-analysis of euglycaemic–hyperinsulinaemic clamp studies. Hum. Reprod..

[55] Condorelli R.A., Calogero A.E., Di Mauro M., La Vignera S. (2017). PCOS and diabetes mellitus: From insulin resistance to altered beta pancreatic function, a link in evolution. Gynecol. Endocrinol..

[56] Lizneva D.V., Gavrilova-Jordan L., Walker W., Azziz R. (2016). Androgen excess: Investigations and management. Best Pr. Res. Clin. Obstet. Gynaecol..

[57] Macut D., Bjekić-Macut J., Rahelić D., Doknić M. (2017). Insulin and the polycystic ovary syndrome. Diabetes Res. Clin. Pr..

[58] Baskind N.E., Balen A.H. (2016). Hypothalamic–pituitary, ovarian and adrenal contributions to polycystic ovary syndrome. Best Pr. Res. Clin. Obstet. Gynaecol..

[59] Ianoşi S., Ianoşi G., Neagoe D., Ionescu O., Zlatian O., Docea A.O., Badiu C., Sifaki M., Tsoukalas D., Tsatsakis A. (2016). Age-dependent endocrine disorders involved in the pathogenesis of refractory acne in women. Mol. Med. Rep..

[60] Moore A.M., Campbell R.E. (2017). Polycystic ovary syndrome: Understanding the role of the brain. Front. Neuroendocr..

[61] Coyle C., Campbell R.E. (2019). Pathological pulses in PCOS. Mol. Cell. Endocrinol..

[62] Ruddenklau A., E Campbell R. (2019). Neuroendocrine Impairments of Polycystic Ovary Syndrome. Endocrinology.

[63] Zhu J., Chen Z., Feng W.-J., Long S.-L., Mo Z.-C. (2019). Sex hormone-binding globulin and polycystic ovary syndrome. Clin. Chim. Acta.

[64] Li Y., Zheng Q., Sun D., Cui X., Chen S., Bulbul A., Liu S., Yan Q. (2018). Dehydroepiandrosterone stimulates inflammation and impairs ovarian functions of polycystic ovary syndrome. J. Cell. Physiol..

[65] Sanchez-Garrido M.A., Tena-Sempere M. (2020). Metabolic dysfunction in polycystic ovary syndrome: Pathogenic role of androgen excess and potential therapeutic strategies. Mol. Metab..

[66] Liu Y., Liu H., Li Z., Fan H., Yan X., Liu X., Xuan J., Feng D., Wei X. (2021). The Release of Peripheral Immune Inflammatory Cytokines Promote an Inflammatory Cascade in PCOS Patients via Altering the Follicular Microenvironment. Front. Immunol..

[67] Zuo T., Zhu M., Xu W. (2016). Roles of Oxidative Stress in Polycystic Ovary Syndrome and Cancers. Oxidative Med. Cell. Longev..

[68] Rudnicka E., Suchta K., Grymowicz M., Calik-Ksepka A., Smolarczyk K., Duszewska A., Smolarczyk R., Meczekalski B. (2021). Chronic Low Grade Inflammation in Pathogenesis of PCOS. Int. J. Mol. Sci..

[69] Shorakae S., Ranasinha S., Abell S., Lambert G., Lambert E., De Courten B., Teede H. (2018). Inter-related effects of insulin resistance, hyperandrogenism, sympathetic dysfunction and chronic inflammation in PCOS. Clin. Endocrinol..

[70] Stepto N.K., Moreno-Asso A., McIlvenna L., A Walters K., Rodgers R.J. (2019). Molecular Mechanisms of Insulin Resistance in Polycystic Ovary Syndrome: Unraveling the Conundrum in Skeletal Muscle?. J. Clin. Endocrinol. Metab..

[71] Mancini A., Bruno C., Vergani E., D′abate C., Giacchi E., Silvestrini A. (2021). Oxidative Stress and Low-Grade Inflammation in Polycystic Ovary Syndrome: Controversies and New Insights. Int. J. Mol. Sci..

[72] Mizgier M., Jarząbek-Bielecka G., Wendland N., Jodłowska-Siewert E., Nowicki M., Brożek A., Kędzia W., Formanowicz D., Opydo-Szymaczek J. (2021). Relation between Inflammation, Oxidative Stress, and Macronutrient Intakes in Normal and Excessive Body Weight Adolescent Girls with Clinical Features of Polycystic Ovary Syndrome. Nutrients.

[73] Zhang R., Liu H., Bai H., Zhang Y., Liu Q., Guan L., Fan P. (2017). Oxidative stress status in Chinese women with different clinical phenotypes of polycystic ovary syndrome. Clin. Endocrinol..

[74] Liu Y., Yu Z., Zhao S., Cheng L., Man Y., Gao X., Zhao H. (2021). Oxidative stress markers in the follicular fluid of patients with polycystic ovary syndrome correlate with a decrease in embryo quality. J. Assist. Reprod. Genet..

[75] Di Segni C., Silvestrini A., Fato R., Bergamini C., Guidi F., Raimondo S., Meucci E., Romualdi D., Apa R., Lanzone A. (2017). Plasmatic and Intracellular Markers of Oxidative Stress in Normal Weight and Obese Patients with Polycystic Ovary Syndrome. Exp. Clin. Endocrinol. Diabetes.

[76] Lai Q., Xiang W., Li Q., Zhang H., Li Y., Zhu G., Xiong C., Jin L. (2018). Oxidative stress in granulosa cells contributes to poor oocyte quality and IVF-ET outcomes in women with polycystic ovary syndrome. Front. Med..

[77] Lu J., Wang Z., Cao J., Chen Y., Dong Y. (2018). A novel and compact review on the role of oxidative stress in female reproduction. Reprod. Biol. Endocrinol..

[78] Uyanikoglu H., Sabuncu T., Dursun H., Sezen H., Aksoy N. (2017). Circulating levels of apoptotic markers and oxidative stress parameters in women with polycystic ovary syndrome: A case-controlled descriptive study. Biomarkers.

[79] Özer A., Bakacak M., Kiran H., Ercan O., Kostu B., Pektas M.K., Kilinç M., Aslan F. (2016). Increased oxidative stress is associated with insulin resistance and infertility in polycystic ovary syndrome. Ginekol. Pol..

[80] Guzmán Hernández E.A., Díaz Portillo S.A., Villafuerte Anaya Ó.C., González Valle M.D.R., Benítez Flores J.D.C., Chávez R.S.M., Galindo G.C., Mondragón L.D.V., Cobos D.S., Guerrero G.A.M. (2020). Renoprotective and Hepatoprotective Effects Of Hippocratea Excelsa On Metabolic Syndrome In Fructose-Fed Rats. Farmacia.

[81] Delitala A., Capobianco G., Delitala G., Cherchi P.L., Dessole S. (2017). Polycystic ovary syndrome, adipose tissue and metabolic syndrome. Arch. Gynecol. Obstet..

[82] Watanabe T., Watanabe-Kominato K., Takahashi Y., Kojima M., Watanabe R. (2017). Adipose Tissue-Derived Omentin-1 Function and Regulation. Compr. Pshysiol..

[83] Dumesic D.A., Abbott D.H., Sanchita S., Chazenbalk G.D. (2020). Endocrine–metabolic dysfunction in polycystic ovary syndrome: An evolutionary perspective. Curr. Opin. Endocr. Metab. Res..

[84] Zeind C.S., Carvalho M.G. (2017). Applied Therapeutics: The Clinical Use of Drugs.

[85] Liu H.-Y., Liu J.-Q., Mai Z.-X., Zeng Y.-T. (2014). A Subpathway-Based Method of Drug Reposition for Polycystic Ovary Syndrome. Reprod. Sci..

[86] Zhang X., Zheng Y., Guo Y., Lai Z. (2019). The Effect of Low Carbohydrate Diet on Polycystic Ovary Syndrome: A Meta-Analysis of Randomized Controlled Trials. Int. J. Endocrinol..

[87] Brennan L., Teede H., Skouteris H., Linardon J., Hill B., Moran L. (2017). Lifestyle and Behavioral Management of Polycystic Ovary Syndrome. J. Women’s Health.

[88] Hakimi O., Cameron L.-C. (2016). Effect of Exercise on Ovulation: A Systematic Review. Sports Med..

[89] Jia L.-Y., Feng J.-X., Li J.-L., Liu F.-Y., Xie L.-Z., Luo S.-J., Han F.-J. (2021). The Complementary and Alternative Medicine for Polycystic Ovary Syndrome: A Review of Clinical Application and Mechanism. Evid.-Based Complement. Altern. Med..

[90] Shen W., Jin B., Pan Y., Han Y., You T., Zhang Z., Qu Y., Liu S., Zhang Y. (2021). The Effects of Traditional Chinese Medicine-Associated Complementary and Alternative Medicine on Women with Polycystic Ovary Syndrome. Evid.-Based Complement. Altern. Med..

[91] Raja-Khan N., Stener-Victorin E., Wu X., Legro R.S. (2011). The physiological basis of complementary and alternative medicines for polycystic ovary syndrome. Am. J. Physiol. Metab..

[92] Zhang Y., Guo X., Ma S., Ma H., Li H., Wang Y., Qin Z., Wu X., Han Y., Han Y. (2021). The Treatment with Complementary and Alternative Traditional Chinese Medicine for Menstrual Disorders with Polycystic Ovary Syndrome. Evid.-Based Complement. Altern. Med..

[93] Shirvani-Rad S., Tabatabaei-Malazy O., Mohseni S., Hasani-Ranjbar S., Soroush A.-R., Hoseini-Tavassol Z., Ejtahed H.-S., Larijani B. (2021). Probiotics as a Complementary Therapy for Management of Obesity: A Systematic Review. Evid.-Based Complement. Altern. Med..

[94] Li Y., Peng C., Cao G., Li W., Hou L. (2018). Tai chi for overweight/obese adolescent and young women with polycystic ovary syndrome: Study protocol for a randomized controlled trial. Trials.

[95] Mohseni M., Eghbali M., Bahrami H., Dastaran F., Amini L. (2021). Yoga Effects on Anthropometric Indices and Polycystic Ovary Syndrome Symptoms in Women Undergoing Infertility Treatment: A Randomized Controlled Clinical Trial. Evid.-Based Complement. Altern. Med..

[96] Thomson R.L., Spedding S., Brinkworth G.D., Noakes M., Buckley J.D. (2013). Seasonal effects on vitamin D status influence outcomes of lifestyle intervention in overweight and obese women with polycystic ovary syndrome. Fertil. Steril..

[97] Legro R.S., Duguech L.M.M. (2017). Pharmacologic Treatment of Polycystic Ovary Syndrome: Alternate and Future Paths. Semin. Reprod. Med..

[98] Ortega I., A Villanueva J., Wong D.H., Cress A.B., Sokalska A., Stanley S.D., Duleba A.J. (2014). Resveratrol potentiates effects of simvastatin on inhibition of rat ovarian theca-interstitial cells steroidogenesis. J. Ovarian Res..

[99] Crandall J.P., Oram V., Trandafirescu G., Reid M., Kishore P., Hawkins M., Cohen H.W., Barzilai N. (2012). Pilot Study of Resveratrol in Older Adults With Impaired Glucose Tolerance. J. Gerontol. Ser. A Boil. Sci. Med Sci..

[100] Rondanelli M., Infantino V., Riva A., Petrangolini G., Faliva M.A., Peroni G., Naso M., Nichetti M., Spadaccini D., Gasparri C. (2020). Polycystic ovary syndrome management: A review of the possible amazing role of berberine. Arch. Gynecol. Obstet..

[101] Naka K.K., Kalantaridou S.N., Kravariti M., Bechlioulis A., Kazakos N., Calis K.A., Makrigiannakis A., Katsouras C.S., Chrousos G.P., Tsatsoulis A. (2011). Effect of the insulin sensitizers metformin and pioglitazone on endothelial function in young women with polycystic ovary syndrome: A prospective randomized study. Fertil. Steril..

[102] Ethinyl Estradiol and Levonorgestrel. https://www.drugs.com/mtm/ethinyl-estradiol-and-levonorgestrel.html.

[103] Mircette® Drug Information. https://www.accessdata.fda.gov/drugsatfda_docs/label/2012/020713s010lbl.pdf.

[104] Diane-35® Drug Information. https://www.bayer.com/sites/default/files/DIANE_35_EN_PI.pdf.

[105] Yasmin® Drug Infromation. https://www.accessdata.fda.gov/drugsatfda_docs/label/2012/021098s019lbl.pdf.

[106] Yaz® Drug Information. https://www.accessdata.fda.gov/drugsatfda_docs/label/2012/021676s012lbl.pdf.

[107] Natazia® Drug Information. https://www.accessdata.fda.gov/drugsatfda_docs/label/2012/022252s001lbl.pdf.

[108] Zahradnik H. (2005). Belara^®^–A Reliable Oral Contraceptive with Additional Benefits for Health and Efficacy in Dysmenorrhoea. Eur. J. Contracept. Reprod. Health Care.

[109] Provera® Drug Information. https://www.accessdata.fda.gov/drugsatfda_docs/label/2007/011839s071lbl.pdf.

[110] Food and Drug Administration (FDA) (2017). GLUCOPHAGE^®^(Metformin Hydrochloride) Tablets. GLUCOPHAGE^®^ XR (Metformin Hydrochloride) Extended-Release Tablets. Label. https://www.accessdata.fda.gov/drugsatfda_docs/label/2017/020357s037s039,021202s021s023lbl.pdf.

[111] Highlights of Prescribing Information-Aldactone® (Spironolactone) Tablets, for Oral Use. https://www.accessdata.fda.gov/drugsatfda_docs/label/2018/012151s075lbl.pdf.

[112] Propecia® Drug Information. https://www.accessdata.fda.gov/drugsatfda_docs/label/2012/020788s020s021s023lbl.pdf.

[113] Clomid® Drug Information. https://www.accessdata.fda.gov/drugsatfda_docs/label/2012/016131s026lbl.pdf.

[114] Femara® Drug Information. https://www.accessdata.fda.gov/drugsatfda_docs/label/2014/020726s027lbl.pdf.

[115] Zocor® Drug Information. https://www.accessdata.fda.gov/drugsatfda_docs/label/2012/019766s085lbl.pdf.

[116] Cassidy-Vu L., Joe E., Kirk J.K. (2016). Role of Statin Drugs for Polycystic Ovary Syndrome. J. Fam. Reprod. Health.

[117] Lipitor® Drug Information. https://www.accessdata.fda.gov/drugsatfda_docs/label/2009/020702s056lbl.pdf.

[118] Food and Drug Administration (FDA) (2011). ACTOS (Pioglitazone Hydrochloride) Tablets for Oral Use. Label. https://www.accessdata.fda.gov/drugsatfda_docs/label/2011/021073s043s044lbl.pdf.

[119] Xu Y., Wu Y., Huang Q. (2017). Comparison of the effect between pioglitazone and metformin in treating patients with PCOS:a meta-analysis. Arch. Gynecol. Obstet..

[120] Jardiance® Drug Information. https://www.accessdata.fda.gov/drugsatfda_docs/label/2020/204629s023lbl.pdf.

[121] Marinkovic-Radosevic J., Berkovic M.C., Kruezi E., Bilic-Curcic I., Mrzljak A. (2021). Exploring new treatment options for polycystic ovary syndrome: Review of a novel antidiabetic agent SGLT2 inhibitor. World J. Diabetes.

[122] Farxiga® Drug Information. https://www.accessdata.fda.gov/drugsatfda_docs/label/2020/202293s020lbl.pdf.

[123] Invokana® Drug Information. https://www.accessdata.fda.gov/drugsatfda_docs/label/2018/204042s027lbl.pdf.

[124] Januvia® Drug Information. https://www.accessdata.fda.gov/drugsatfda_docs/label/2012/021995s019lbl.pdf.

[125] Abdalla M.A., Deshmukh H., Atkin S., Sathyapalan T. (2021). The potential role of incretin-based therapies for polycystic ovary syndrome: A narrative review of the current evidence. Ther. Adv. Endocrinol. Metab..

[126] Victoza® Drug Information. https://www.accessdata.fda.gov/drugsatfda_docs/label/2017/022341s027lbl.pdf.

[127] Cena H., Chiovato L., E Nappi R. (2020). Obesity, Polycystic Ovary Syndrome, and Infertility: A New Avenue for GLP-1 Receptor Agonists. J. Clin. Endocrinol. Metab..

[128] Ozempic® Drug Information. https://www.accessdata.fda.gov/drugsatfda_docs/label/2017/209637lbl.pdf.

[129] Byetta® Drug Information. https://www.accessdata.fda.gov/drugsatfda_docs/label/2009/021773s9s11s18s22s25lbl.pdf.

[130] Cetylev® Drug Information. https://www.accessdata.fda.gov/drugsatfda_docs/label/2016/207916s000lbl.pdf.

[131] Sandhu J.K., Waqar A., Jain A., Joseph C., Srivastava K., Ochuba O., Alkayyali T., Ruo S.W., Poudel S. (2021). Oxidative Stress in Polycystic Ovarian Syndrome and the Effect of Antioxidant N-Acetylcysteine on Ovulation and Pregnancy Rate. Cureus.

[132] Tehran University of Medical Sciences The Effect of Astaxanthin on Oxidative Stress Indices in Patients With Polycystic Ovary Syndrome. https://ClinicalTrials.gov/show/NCT03991286.

[133] Azienda di Servizi alla Persona di Pavia Berberine and Polycystic Ovary Syndrome. https://ClinicalTrials.gov/show/NCT04932070.

[134] Woman’s, AstraZeneca EQW, DAPA, EQW/DAPA, DAPA/MET ER and PHEN/TPM ER in Obese Women With PolycysticOvary Syndrome (PCOS). https://ClinicalTrials.gov/show/NCT02635386.

[135] Biosearch S.A. Evaluation of the Mixture Myoinositol:D-Chiro-Inositol 3.6:1 in Women With Polycystic Ovary Syndrome. https://ClinicalTrials.gov/show/NCT03201601.

[136] Hull University Teaching Hospitals NHS Trust Empagliflozin vs. Metformin in PCOS. https://ClinicalTrials.gov/show/NCT03008551.

[137] Javed Z., Papageorgiou M., Deshmukh H., Rigby A.S., Qamar U., Abbas J., Khan A.Y., Kilpatrick E.S., Atkin S.L., Sathyapalan T. (2019). Effects of empagliflozin on metabolic parameters in polycystic ovary syndrome: A randomized controlled study. Clin. Endocrinol..

[138] RenJi Hospital Research of Exenatide for Overweight/Obese PCOS Patients With IGR. https://ClinicalTrials.gov/show/NCT03352869.

[139] Tao T., Zhang Y., Zhu Y.-C., Fu J.-R., Wang Y.-Y., Cai J., Ma J.-Y., Xu Y., Gao Y.-N., Sun Y. (2021). Exenatide, Metformin, or Both for Prediabetes in PCOS: A Randomized, Open-label, Parallel-group Controlled Study. J. Clin. Endocrinol. Metab..

[140] AGUNCO Obstetrics and Gynecology Centre, Hospital Juarez de Mexico Myo-inositol, Alpha-Lactalbumin and Folic Acid Treatment in PCOS. https://ClinicalTrials.gov/show/NCT04645745.

[141] Lo.Li.Pharma s.r.l Improved Effects of MI Plus Alpha-LA in PCOS. https://ClinicalTrials.gov/show/NCT03422289.

[142] Oliva M.M., Buonomo G., Calcagno M., Unfer V. (2018). Effects of myo-inositol plus alpha-lactalbumin in myo-inositol-resistant PCOS women. J. Ovarian Res..

[143] Pharmarte srl Myoinositol Plus L-Tyrosine, Selenium and Chromium in PCOS. https://ClinicalTrials.gov/show/NCT03673995.

[144] Medical University of Vienna Micronutrient Supplementation in PCO-Syndrome. https://ClinicalTrials.gov/show/NCT03306745.

[145] Hager M., Nouri K., Imhof M., Egarter C., Ott J. (2019). The impact of a standardized micronutrient supplementation on PCOS-typical parameters: A randomized controlled trial. Arch. Gynecol. Obstet..

[146] Universitaire Ziekenhuizen Leuven The Gut Microbiome in Women With Polycystic Ovary Syndrome. https://ClinicalTrials.gov/show/NCT03642600.

[147] Ain Shams University L-Carnitine and Clomiphene Citrate for Induction of Ovulation in Women With Polycystic Ovary Syndrome. https://ClinicalTrials.gov/show/NCT03476356.

[148] University A. Adding L-Carnitine to Clomiphene Citrate for Induction of Ovulation in Women with Polycystic Ovary Syndrome. https://ClinicalTrials.gov/show/NCT03630341.

[149] Woman’s, A/S, N.N Liraglutide 3mg (Saxenda) on Weight, Body Composition, Hormonal and Metabolic Parameters in Obese Women with PCOS. https://ClinicalTrials.gov/show/NCT03480022.

[150] University A.S. NAC in CC Resistant PCOS After LOD. https://ClinicalTrials.gov/show/NCT02775734.

[151] Shiraz University of Medical Sciences Effects of Cyproterone Compound-spironolactone, Metformin and Pioglitazone on Inflammatory Markers in PCOS. https://ClinicalTrials.gov/show/NCT02689843.

[152] Peshawar K.M.U. Treatment with Metformin and Combination of Metformin and Pioglitazone in Polycystic Ovarian Syndrome. https://ClinicalTrials.gov/show/NCT03117517.

